# Plastid Genome Evolution in the Early-Diverging Legume Subfamily Cercidoideae (Fabaceae)

**DOI:** 10.3389/fpls.2018.00138

**Published:** 2018-02-08

**Authors:** Yin-Huan Wang, Susann Wicke, Hong Wang, Jian-Jun Jin, Si-Yun Chen, Shu-Dong Zhang, De-Zhu Li, Ting-Shuang Yi

**Affiliations:** ^1^Key Laboratory for Plant Diversity and Biogeography of East Asia, Kunming Institute of Botany, Chinese Academy of Sciences, Yunnan, China; ^2^Germplasm Bank of Wild Species, Kunming Institute of Botany, Chinese Academy of Sciences, Yunnan, China; ^3^Kunming College of Life Sciences, University of Chinese Academy of Sciences, Beijing, China; ^4^Institute for Evolution and Biodiversity, University of Münster, Münster, Germany

**Keywords:** inversion, isomeric plastomes, IR expansion/contraction, plastome, variation, Cercidoideae, Fabaceae

## Abstract

The subfamily Cercidoideae is an early-branching legume lineage, which consists of 13 genera distributed in the tropical and warm temperate Northern Hemisphere. A previous study detected two plastid genomic variations in this subfamily, but the limited taxon sampling left the overall plastid genome (plastome) diversification across the subfamily unaddressed, and phylogenetic relationships within this clade remained unresolved. Here, we assembled eight plastomes from seven Cercidoideae genera and conducted phylogenomic-comparative analyses in a broad evolutionary framework across legumes. The plastomes of Cercidoideae all exhibited a typical quadripartite structure with a conserved gene content typical of most angiosperm plastomes. Plastome size ranged from 151,705 to 165,416 bp, mainly due to the expansion and contraction of inverted repeat (IR) regions. The order of genes varied due to the occurrence of several inversions. In *Tylosema* species, a plastome with a 29-bp IR-mediated inversion was found to coexist with a canonical-type plastome, and the abundance of the two arrangements of isomeric molecules differed between individuals. Complete plastome data were much more efficient at resolving intergeneric relationships of Cercidoideae than the previously used selection of only a few plastid or nuclear loci. In sum, our study revealed novel insights into the structural diversification of plastomes in an early-branching legume lineage, and, thus, into the evolutionary trajectories of legume plastomes in general.

## Introduction

Chloroplast genomes (plastomes) of photosynthetic angiosperms usually are highly conserved regarding their overall gene content (115–160 genes) and order and GC content (34–40%). They often present a quadripartite structure that consists of a pair of large inverted repeats (IRs; usually around 25 kb, but can vary from 7 to 88 kb) separated by large and small single copy regions (LSC of ca. 80 kb length and SSC of ca. 20 kb, respectively) (Jansen and Ruhlman, 2012; Ruhlman and Jansen, 2014). The large IRs of the plastome are hypothesized to contribute to plastome stabilization, because their absence often coincides with additional severe changes of gene order (Palmer and Thompson, 1982), although causation remains unclear. With the advent of next-generation sequencing, complete plastome sequencing has increased dramatically. We are becoming more and more aware of an increasing number of plastome rearrangements also in photosynthetic angiosperms that retain a quadripartite structure, like in Campanulaceae (Cosner et al., 2004; Haberle et al., 2008), Geraniaceae (Palmer et al., 1987; Chumley et al., 2006; Guisinger et al., 2011; Weng et al., 2014), or Oleaceae (Lee et al., 2007).

The legume family (Fabaceae) is notable for its departures from the typical plastome structure, of which several rearrangements are of phylogenetic relevance. Plastome size in legumes varies greatly because of either expansion, contraction or loss of the IR. Smaller plastomes characterize species of the inverted repeat-lacking clade (IRLC), which have lost the IR (Wojciechowski et al., 2000). In contrast, larger plastomes are typical of species in the inverted repeat-expanding clade (IREC) that have IRs expanding into the SSC by ca. 13 kb (Dugas et al., 2015; Wang et al., 2017a). The loss of two housekeeping genes, namely the translation initiation factor (*infA*) and the ribosomal protein L22 (*rpl22*), is shared among all legumes (Gantt et al., 1991; Magee et al., 2010). Other genes, such as *accD, clpP, psaI, rpl33, rps16*, and *ycf4*, have been functionally lost in various legume lineages (Keller et al., 2017). In addition, group IIA-introns have been lost from *clpP, rpl2*, and *rps12* in various legume lineages (Doyle et al., 1995; Jansen et al., 2008; Dugas et al., 2015; Wang et al., 2017a). Many of these unusual plastome features of legumes known so far are restricted to papilionoids and mimosoids, and modifications of the “normal” angiosperm plastome structure (a unique 7.5-kb inversion and 5-kb IR expansion into SSC) have been reported only in *Tylosema esculentum* of Cercidoideae (Kim and Cullis, 2017).

Plastome inversions are common in papilionoids. Except for a few of early diverging lineages of papilionoids, members of this subfamily typically share a 50-kb inversion in the LSC (Doyle et al., 1996). A 78-kb inversion characterizes the subtribe Phaseolinae of Phaseoleae (Bruneau et al., 1990), whereas in *Robinia* a 39-kb inversion is known (Schwarz et al., 2015). Inversions of 23, 24, or 36 kb have been reported in different taxa of the Genistoid clade (Martin et al., 2014; Choi and Choi, 2017; Feng et al., 2017; Keller et al., 2017), and multiple inversions have been detected in IRLC-legumes (Milligan et al., 1989; Cai et al., 2008; Sabir et al., 2014; Sveinsson and Cronk, 2014; Lei et al., 2016). However, only two inversions have been reported in other legumes, including the aforementioned 7.5-kb inversion from *T. esculentum* (Kim and Cullis, 2017) and a 421-bp inversion from a mimosoid species (Wang et al., 2017a). The 36-kb and 39-kb inversions of some genistoids and *Robinia* mentioned above, respectively, are both located between a pair of 29-bp short inverted repeats situated in the 3′-ends of two *trnS* genes. Inverted repeats are thought to contribute to inversions by mediating intramolecular recombination that may result in the formation of isomers. The most typical example of such isomers is illustrated by the relative orientation of single copy (SC) regions existing in a single plant as demonstrated by Palmer (1983). Besides, Stein et al. (1986) predicted a universal existence of isomeric plastomes in all plastomes with typical IRs. Some isomeric plastomes caused by small inverted repeats other than the primary IRs have been reported in several conifers (Tsumura et al., 2000; Wu et al., 2011; Yi et al., 2013; Guo et al., 2014; Qu et al., 2017b). However, it remains unknown whether the 29-bp IRs in legume plastomes could also mediate isomers.

Cercidoideae is one of six recently recognized subfamilies of Fabaceae (LPWG, 2017) and probably represents the first-branching legume lineage (Bruneau et al., 2001; Herendeen et al., 2003). This subfamily consists of ca. 335 species in 13 genera that are distributed in tropical and warm temperate regions of the Northern Hemisphere (Clark et al., 2017; LPWG, 2017). Some of its species are of economic value to humans. For instance, several species of *Barklya, Bauhinia, Cercis, Griffonia, Phanera, Piliostigma*, and *Tylosema* are valued for the production of foods, timbers, dyes, or ropes, or they find application as medicinal and ornamental plants, or even as coffee substitutes (Lewis et al., 2005). Phylogenetically, intergeneric relationships of Cercidoideae remain unresolved in previous phylogenetic studies (Bruneau et al., 2008; Sinou et al., 2009; LPWG, 2013, 2017). Clarifying relationships in Cercidoideae will facilitate many aspects of studies on this economically important group, and contribute to elucidating the evolutionary trajectory of plastid genome evolution in legumes in general. Given plastome variations having been found in only four published species, it is likely that more divergent plastomes hide in Cercidoideae.

Here, we present an analysis of eight newly sequenced plastomes of Cercidoideae. We complement our dataset with four more species from three genera of this subfamily and 45 other legumes to reconstruct the phylogeny of Cercidoideae based on 77 protein-coding genes, 136 intergenic spacers, and 19 introns. Our comparative plastome analysis involving examinations of IR boundary shifts, inversions and locally collinear blocks (LCB), and the existence of isomeric plastomes uncovers unique plastome features and illustrates the variation of plastomes in this clade. Finally, a critical review of plastome structures across the legume family sheds further light on the mechanisms of plastome evolution in Fabaceae.

## Materials and Methods

### Plant Sampling

For the plastome analysis, we sampled fresh or silica gel-dried leaves of eight species representing seven genera of the subfamily Cercidoideae. Of these, plastomes of genera *Barklya, Bauhinia, Griffonia, Lysiphyllum, Piliostigma*, and *Schnella* were sequenced for the first time. To verify the existence of isomeric plastomes, we isolated total genomic DNA of three additional individuals of two *Tylosema* species. Supplementary Table S1 summarizes voucher information and material type for sampled plants.

### Chloroplast DNA Extraction and Sequencing

Total genomic DNA was isolated with a modified CTAB (Cetyl Trimethyl Ammonium Bromide) method described in Yang et al. (2014). For species from which DNA was obtained from fresh leaves, chloroplast DNA (cpDNA) was amplified using long-range PCR (LPCR) with fifteen primer pairs described in Zhang et al. (2016). DNA extracts and cpDNA-amplicons were fragmented for short-insert, paired-end (PE) library construction and sequenced on either an Illumina HiSeq 2000/2500 or X-Ten instrument at Beijing Genomics Institute (BGI, Shenzhen, China), or at the Plant Germplasm and Genomics Center, Kunming Institute of Botany, Chinese Academy of Sciences (KIB, CAS, Kunming, China), respectively.

### Plastome Assembly and Annotation

All raw sequence data from LPCR-based plastome enrichment was quality-checked using the NGS QC Tool Kit (Patel and Jain, 2012) with default parameters. High-quality PE reads were *de novo* assembled into contigs using *CLC Genomics Workbench* v.8.5.1 with a k-mer of 63 and an automatic bubble size. We retained only contigs with a minimum length of 1 kb and aligned these with *Haematoxylum brasiletto* (NC_026679) as reference employing nucleotide BLAST (Altschul et al., 1990) at default search parameters. Then, the most probable order of the aligned contigs was determined according to the reference plastome, and the gaps between the contigs were filled by mapping the raw reads to the reference plastome. For shotgun-sequenced genomic DNA, raw reads were filtered and assembled into contigs using GetOrganelle.py^1^ with the plastome of *H. brasiletto* as the reference. Contigs were then connected with the help of Bandage Ubuntu dynamic v.080 (Wick et al., 2015) and manual correction where necessary.

Annotation of the plastomes was performed in DOGMA (Wyman et al., 2004), coupled with manual corrections in Geneious v.9.0.2 (Biomatters, Inc.). Protein coding genes were double-checked by finding open reading frames using the Find ORFs function in Geneious v.9.0.2. We used the online tRNAscan-SE service (Schattner et al., 2005) to improve the identification of tRNA genes. Physical maps of all sequenced plastomes were prepared with OrganellarGenomeDRAW v.1.2 (Lohse et al., 2013), and are enclosed here as Supplementary Figure S1. To detect the number of matched reads and the depth of coverage, raw reads were remapped to the assembled plastomes with Bowtie2 (Langmead and Salzberg, 2012), implemented in Geneious v.9.0.2. We used the End-to-End alignment type and Medium Sensitivity/Fast preset, and adjusted the maximum insert size to 800 bp; sequences remained untrimmed before mapping. The final annotated plastomes are deposited in GenBank under accession numbers MF135594–MF135601 (**Table 1**).

**Table 1:** Summary of plastome features of sampled Cercidoideae species.

	*Adenolobus garipensis*	*Barklya syringifolia*^∗^	*Bauhinia acuminata*^∗^	*Cercis Canadensis*	*Cercis glabra*	*Griffonia simplicifolia*^∗^	*Lysiphyllum sp. 1*^∗^	*Lysiphyllum sp. 2*^∗^	*Piliostigma thonningii*^∗^	*Schnella trichosepala*^∗^	*Tylosema esculentum*	*Tylosema fassoglensis*^∗^
GenBank accession	KY806280	MF135594	MF135595	KF856619	KY806281	MF135596	MF135601	MF135597	MF135598	MF135599	KX792933	MF135600
Entire plastome size (bp)	151,705	158,740	155,548	158,995	159,181	157,909	154,055	153,990	165,416	159,722	161,537	161,803
LSC size (bp)	83,279	88,585	86,279	88,118	88,240	87,647	86,976	86,859	71,912	87,791	86,111	86,107
SSC size (bp)	18,444	17,851	18,141	19,621	19,691	18,788	18,307	18,305	18,098	13,757	13,632	13,666
IR size (bp)	24,991	26,152	25,564	25,628	25,625	25,737	24,386	24,413	37,703	29,087	30,897	31,015
No. unique genes	111	111	111	111	111	111	111	111	111	111	111	111
No. unique PCGs/In IR	77/5	77/4	77/5	77/5	77/5	77/5	77/4	77/4	77/19	77/5	77/6	77/6
No. unique tRNAs/ In IR	30/7	30/7	30/7	30/7	30/7	30/7	30/7	30/7	30/7	30/7	30/7	30/7
No. unique rRNAs/ In IR	4/4	4/4	4/4	4/4	4/4	4/4	4/4	4/4	4/4	4/4	4/4	4/4
No. unique genes with introns/two introns	12/3	12/3	11/3	12/3	12/3	12/3	12/3	12/3	12/3	12/3	12/3	12/3
GC content (%)	36.9	36.4	36.5	36.2	36.2	36.3	36.6	36.6	36.4	36.2	36.0	36.0
Mean coverage (×)	1895.3	2,030.2	1,915.5	NA	2,507.8	313.6	1,438.5	1,527.0	1,583.4	658.7	NA	1,023.4
Number of inversions	0	0	0	0	0	1	0	0	1	0	1	2

*Asterisk marks species sequenced in this study.*

### Analysis of Plastome Rearrangements and Inversions

To detect the breakpoints of inversions in the plastomes of *Griffonia simplicifolia, Piliostigma thonningii, Tylosema esculentum*, and *T. fassoglensis*, and to identify locally collinear blocks (LCBs) in plastomes of all sampled legumes (see “Phylogeny reconstruction” in Section “Materials and Methods” and Supplementary Table S2), we performed a whole-plastome alignment using Mauve v.2.3.1 (Darling et al., 2010), implemented in Geneious v.9.0.2. To this end, we used the progressiveMauve algorithm with both the seed weight and minimum LCB score being calculated automatically. To detect the breakpoints of inversions in those four species, the plastome of *Cercis glabra*, which has a typical angiosperm plastome organization, was used as the reference in Mauve alignments. For the Mauve alignment of all sampled legumes, species were ordered alphabetically. Because IRLC legumes all lost the IR_A_ in their plastomes, the IR_A_ of plastomes was removed for each species outside the IRLC.

### Analysis of Isomeric Plastomes

We found a 38-kb inversion between a pair of 29-bp IRs in the 3′-ends of *trnS^GCU^* and *trnS^GGA^* that was absent from the plastome of the previously published *Tylosema* species (Kim and Cullis, 2017). We pursued two approaches to explore whether a plastome with this inversion coexists with a canonical plastome in *Tylosema fassoglensis* and other *Tylosema* species: Firstly, we used Bowtie2 (as before) to map raw PE reads of *T. fassoglensis* to the four regions corresponding to the breakpoints of the inversion. For convenience, we here defined the plastome with the 38-kb inversion as IPWI (isomeric plastome with inverted arrangement), whereas the plastome with its reverse-complement (canonical) orientation as IPWC (isomeric plastome with canonical arrangement). Accordingly, *psbI*-*trnS^GGA^*-*ycf3* and *trnG^UCC^*-*trnS^GCU^*-*rps4* both were captured from IPWI, whereas *psbI*-*trnS^GCU^*-*trnG^UCC^* and *ycf3*-*trnS^GGA^*-*rps4* were captured from IPWC. Secondly, we performed a PCR assay with specially designed primer pairs that target all four breakpoint regions of the isomeric plastomes. In so doing, we included three additional individuals of *Tylosema* (Supplementary Table S1) to investigate the universality of the isomeric plastomes in this genus. Each of the 25.5 μL PCR reaction mixture contained 1 μL total genomic DNA (ca. 100 ng/μL) as the template, 0.5 μL each of the forward and reverse primers (10 μmol/L), 12.5 μL Tiangen 2× Taq PCR MasterMix, and 11 μL double-distilled water. To account for potentially different qualities of the template DNAs as well as the possibility of the non-universality of the primer pairs, we used different primer pairs and PCR conditions in our screening, as detailed in Supplementary Table S3.

### Phylogenetic Reconstructions

To reconstruct the phylogenetic relationships among Cercidoideae and other legumes, we complemented our data set of eight Cercidoideae species with 49 previously published legume plastomes (Kato et al., 2000; Saski et al., 2005; Guo et al., 2007; Jansen et al., 2008; Magee et al., 2010; Kazakoff et al., 2012; Sabir et al., 2014; Sveinsson and Cronk, 2014; Dugas et al., 2015; Lei et al., 2016; Choi and Choi, 2017; Donkpegan et al., 2017; Feng et al., 2017; Kim and Cullis, 2017; Wang et al., 2017a,b and *Cadellia pentastylis* (Surianaceae; Li et al., unpublished data) as outgroups (Supplementary Table S2). Protein coding (CDS) and non-genic (intergenic [IGS] and intron) sequences were extracted and aligned to generate original CDS, IGS and intron alignments, respectively, using MAFFT v.7.271 (Katoh and Standley, 2013) with default parameters. Ambiguously aligned sites in all these alignments were removed using GBLOCKS v.0.91b (Castresana, 2000; Talavera and Castresana, 2007) with default parameters, except that all gap positions were allowed. All original alignments and the GBLOCKS-edited versions were concatenated separately to generate supermatrices. Four more supermatrices of original and GBLOCKS-edited alignments for non-genic regions (IGS + intron, hereafter: NGS) and all plastome regions (CDS + IGS + intron) were then obtained by concatenating the corresponding supermatrices. These datasets were subjected to maximum likelihood (ML) phylogenetic reconstructions using RAxML-HPC v.8.2.4 (Stamatakis, 2014) with the GTR-CAT substitution model and 1,000 replicates of rapid bootstrap.

## Results

### Organization of Cercidoideae Plastomes

Due to differences regarding both the plant materials and the experimental procedures, the average plastome coverage varied significantly from 313.6× to 2030.2× (**Table 1**). While the total plastome sizes, including their respective LSC, SSC, and IR regions, differ considerably, we observed only marginal variation in GC contents (36.0 to 36.6%). Plastome size among the sampled Cercidoideae species ranges from 151,705 bp in *Adenolobus garipensis* to 165,416 bp in *Piliostigma thonningii*. The length of IR ranges from 24,386 bp in *Lysiphyllum* sp. 1 to 37,703 bp in *P. thonningii*. This followed the substantial length variation for LSC from 71,912 bp in *P. thonningii* to 88,585 bp in *Barklya syringifolia*. The length of the SSC also varies substantially, ranging from 13,632 bp in *Tylosema esculentum* to 19,691 bp in *Cercis glabra*.

### Plastome Rearrangement

All sampled plastomes of Cercidoideae exhibit a typical quadripartite structure and a conserved gene content; only *Bauhinia acuminata* has lost the *rpl2* intron (Supplementary Figure S1). The plastid *accD* genes in species of *Barklya, Lysiphyllum, Schnella*, and *Tylosema* apparently lack 260-714 bp at their respective 5′-ends, and the *matK* genes of *Tylosema* are 131-bp shorter at their 5′-ends compared with other Cercidoideae species. However, both genes retain intact open reading frames (ORFs).

**FIGURE 1:**
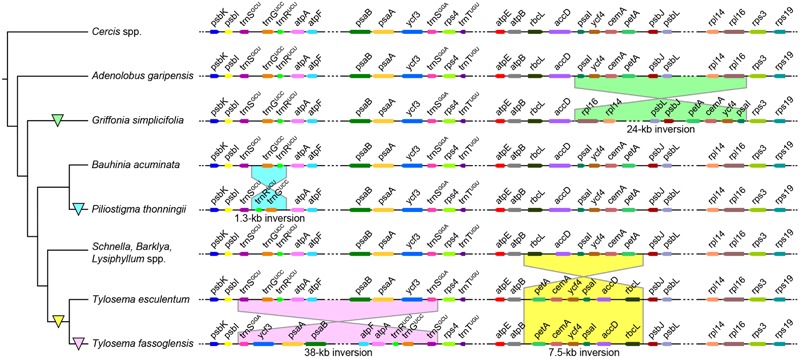
Plastome inversions in Cercidoideae. *Griffonia, Piliostigma*, and *Tylosema* show independent inversions in their LSCs compared with other closely related Cercidoideae. The partial plastome maps **(right)** are not drawn to scale, and the tree topology **(left)** is depicted according to our phylogenic reconstructions based on the combined plastid coding regions (see Results). Colored triangles on branches demarcate the evolutionary origin of the inversion shown to the right.

Unlike gene content, gene order differs notably between species due to the occurrence of several inversions (**Figure 1**). The plastome of *Griffonia simplicifolia* has a 24-kb inversion from its *rpl16* gene to *psaI*, resulting in the adjacencies of *accD* with *psaI* and *rpl16* with *rps3*. In contrast, a 1.3-kb inversion from *trnR^UCU^* and *trnG^UCC^*, which results in *trnS^GCU^* neighboring *trnR^UCU^* and *trnG^UCC^* being adjacent to *atpA*, characterizes the plastome of *P. thonningii*. *Tylosema fassoglensis* has two inversions in its plastome, one of which is a 7.5-kb inversion spanning from *rbcL* to *petA*, thus resulting in the colocalizations of *atpB* with *petA* and *rbcL* with *psbJ* – a gene order seen in *T. esculentum*, too. The second inversion of 38 kb in size lies between the 29-bp IRs at the 3′ -ends of *trnS^GCU^* and *trnS^GGA^* genes, and, in consequence, *psbI* is positioned adjacent to *trnS^GGA^* and *trnS^GCU^* neighbors *rps4*.

**FIGURE 2:**
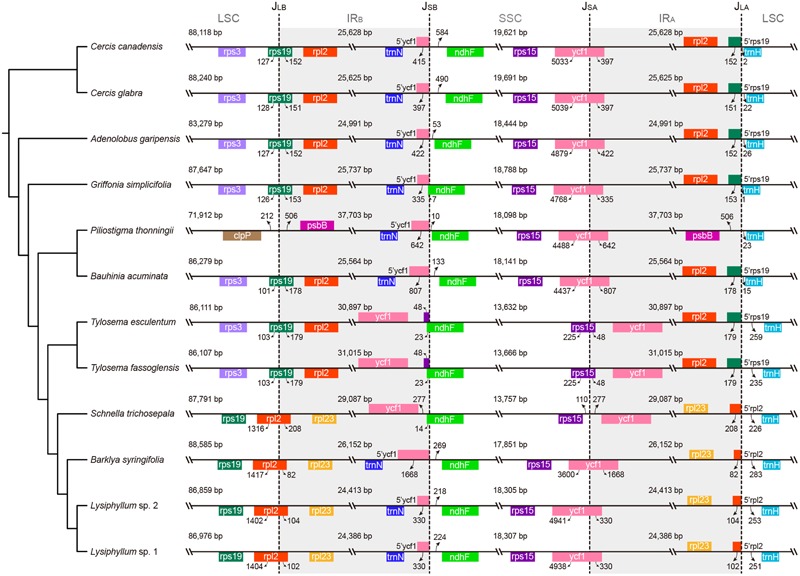
IR/SC junctions in Cercidoideae plastomes. Junctions from IRs into single-copy regions are depicted for all Cercidoideae, which are arranged according to their phylogenetic placement. J_LB_, J_SB_, J_SA_, and J_LA_ refer to the junctions of LSC/IR_B_, SSC/IR_B_, SSC/IR_A_, and LSC/IR_A_, respectively. Genes are indicated by colored boxes above (encoded on the plus strand) and below (minus strand) the horizontal lines. The tree topology (left) is modified from our reconstructed phylogeny (as in **Figure 1**; see Results).

The plastomes of *Adenolobus, Bauhinia, Cercis*, and *Griffonia* have a conserved IR length, ranging from 24,991 (*A. garipensis*) to 25,737 bp (*G. simplicifolia*) (**Figure 2** and Supplementary Figure S1). The locations of IR-SC junctions of these plastomes are also similar to other canonical angiosperm plastomes. Specifically, the LSC/IR_B_ junction (J_LB_) lies within the *rps19* gene, which leads to some length variation regarding the duplicated 3’-ends of this gene (from 151 bp in *Cercis glabra* to 178 bp in *Bau. acuminate*) at the border of the IR_A_/LSC junction (J_LA_) between *rpl2* (IR_A_) and *trnH* (LSC). The position of the IR_A_/SSC junction (J_SA_) is within the *ycf1*. Hence, the duplicated 3′-ends of *ycf1* range from 335 (*G. simplicifolia*) to 807 bp (*Bau. acuminata*) at the border of the IR_B_/SSC junction (J_SB_) between *trnN* (IR_B_) and *ndhF* (SSC). The plastomes of *Bar. syringifolia, Lysiphyllum* sp. 1, *Lysiphyllum* sp. 2, and *Schnella trichosepala* uniformly exhibit a contraction of their IRs. Their LSCs are narrowed by ca. 1.6 kb due to a shift of the J_LB_ into the 3′-exon of *rpl2* through which 82 (*Bar. syringifolia*) to 208 bp (*S. trichosepala*) of this gene’s 3′-ends is duplicated in the IR_A_. Among these taxa, *S. trichosepala* also displays an expansion of approximately 5 kb on the opposite end of its IR, which now contains the entire *ycf1* gene, and the J_SB_ lies in the 5′-end of *ndhF* (14 bp are duplicating in IR_A_). *Tylosema esculentum* and *T. fassoglensis* similarly expanded their IRs to contain intact *ycf1*; J_SB_ and J_SA_ are accordingly shifted into *ndhF* and *rps15*, respectively. The IRs of *Bar. syringifolia* expand by ca. 1.3 kb into the SSC but the IR/SSC junction remains within the *ycf1* coding region. With a gain of 12 kb in size, *P. thonningii* shows the most extreme IR expansion, leading to the duplication of both *rps19* and 13 other genes spanning from *rps3* to *psbB*, and the J_LB_ lies in the intergenic spacer of *clpP* and *psbB*.

### Isomeric Plastomes in *Tylosema* Species

**FIGURE 3:**
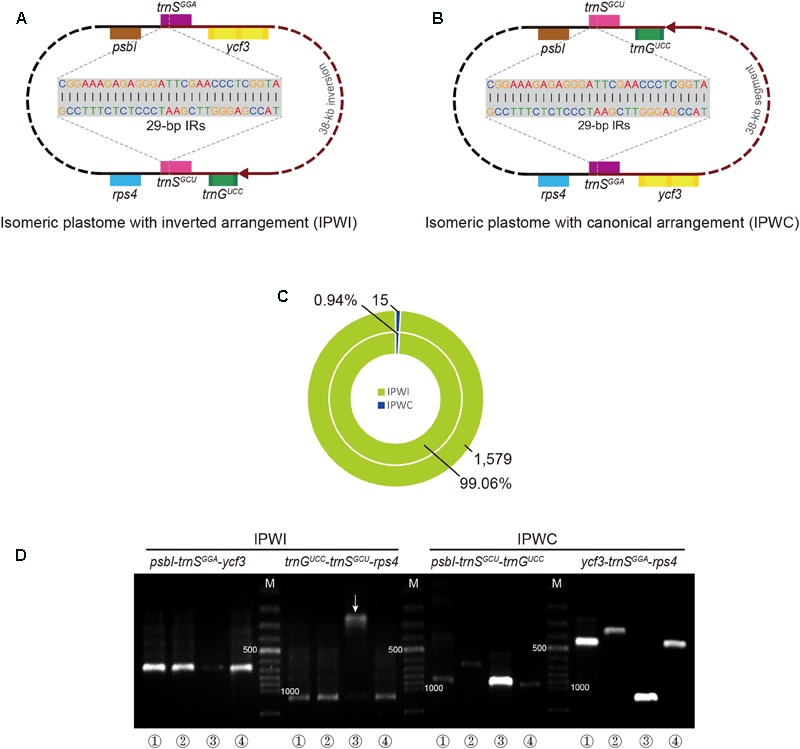
Isomeric plastomes in *Tylosema* species. **(A)** Gene adjacency at the inversion breakpoints in the IPWI-type plastome. **(B)** Gene adjacency at inversion breakpoints in the IPWC-type plastome. **(C)** The results of our read-mapping analysis are depicted as numbers of paired-end reads spanning the 29-bp IR in the outer (total number of reads) and inner circles (proportion of matched reads to total mapped reads). **(D)** PCR amplicons of four breakpoint regions in four individuals of *Tylosema* (1, *T. fassoglensis*; 2, *T. fassoglensis* 1; 3, *T. fassoglensis* 2; 4, *T. esculentum*). The white arrow highlights an abnormal result that may be caused by poor primer specificity. Red lines in **A** and **B** refer to the 38-kb inverted segments. “M” in **D** refers to a 100 bp plus DNA ladder, with “500” and “1000” indicating fragment lengths of 500 and 1 kb, respectively.

A schematic of the endpoint location of isomeric plastomes is depicted in **Figures 3A,B**. According to the read-mapping results of the putative *T. fassoglensis* isomers, 1,579 reads of the over 15 million PE reads obtained by sequencing span the 29-bp IRs in the IPWI orientation, whereas only 15 sequences cover the 29-bp IRs in the IPWC type. Consequently, the frequency of the IPWI and IPWC can be assumed as 99.06% and 0.94%, respectively (**Figure 3C**). Details regarding the statistics of the read-mapping results are presented in Supplementary Table S4. Using PCR validations, the coexistence of IPWI and IPWC was demonstrated to occur also in other three individuals of *Tylosema* (**Figure 3D**). In general, gel electrophoresis revealed fragments for IPWI of *T. fassoglensis, T. fassoglensis* 1, and *T. esculentum* to be much brighter, i.e., more abundant, than in *T. fassoglensis* 2, while that for IPWC normally are much fainter. In addition, in *T. fassoglensis, T. fassoglensis* 1, and *T. esculentum*, the band expected for IPWI appears much brighter than that for IPWC, except for its *ycf3*-trnS^GGA^-*rps4* region, which may be a result of high primer specificity.

### Plastid Phylogeny of Cercidoideae

Our phylogenetic analyses supported the monophyly of Cercidoideae with strong bootstrap support (BS) of 100% and contributed to clarifying intergeneric relationships (Supplementary Figure S2). *Cercis* is well-resolved as the first-branching lineage (BS = 100%), and *Adenolobus* is confidently placed as sister to the remaining Cercidoideae species (BS = 100%). *Bauhinia* and *Piliostigma* are strongly supported as sister clades (BS ≥ 94%). *Schnella, Barklya*, and *Lysiphyllum* form a clade with BS = 100%. However, our different data matrices produced conflicting relationships among *Griffonia* (G), the *Bauhinia* + *Piliostigma* (BP) clade, and the lineage containing the remaining species from a clade of *Barklya* + *Lysiphyllum* + *Schnella* + *Tylosema*, hereafter: BLST). The matrices of original and GBLOCKS-edited CDS and all markers support *Griffonia* as sister to BP + BLST clade, while other matrices resolve *Griffonia* together with *Bauhinia* and *Piliostigma* as sister to BLST. Incongruence was also found among *Schnella, Barklya* and *Lysiphyllum* in different matrices, but *Schnella* as sister to *Barklya* and *Lysiphyllum* was the most strongly supported relationship.

## Discussion

### Structural Diversity of Plastomes in Cercidoideae

Our plastid genome analyses reveal various structural variations in Cercidoideae, including several inversions, shifts of IR-SC junction, and intron losses. Inversions and IR boundary shifts represent essential mechanisms for plastome rearrangements, which contribute to the structural diversification of plant plastomes (Wicke et al., 2011; Jansen and Ruhlman, 2012). Our study thus adds new results, because legume plastomes outside the Papilionoideae subfamily have long been considered to be conserved regarding their structure and gene content (Schwarz et al., 2015). We now can show that the evolutionary stasis of angiosperm plastomes breaks up already in early-branching lineages of Fabaceae, even though the two early-diverging Cercidoideae genera show no departures from the typical angiosperm plastome organization (Wang et al., 2017b). Major IR expansions and contractions plus some other structural variations such as inversions, gene duplications, and intron losses were also reported for mimosoids recently (Dugas et al., 2015; Wang et al., 2017a).

Inversions of over 1 kb in length are typical for papilionoid plastomes but rarely encountered in other legumes. No large inversions occur in plastomes of Caesalpinioideae (including mimosoids) and Detarioideae, and the unique 7.5-kb inversion has been identified in only one Cercidoideae species, as reported recently (Kim and Cullis, 2017). By analyzing additional genera of Cercidoideae, we here discover that a 7.5-kb inversion is restricted to examined species of *Tylosema* instead of representing a synapomorphy for the *Bauhinia* s.l. group as speculated by Kim and Cullis (2017). We also discover three more inversions in Cercidoideae, one of which, the 38-kb inversion in *Tylosema fassoglensis*, appears to be directly mediated by a pair of 29-bp IRs, resembling the situation of the 36 and 39-kb inversions in Papilionoideae. The other inversions may be promoted by dispersed repeats in the breakpoint regions through intermolecular recombination (Jansen and Ruhlman, 2012). These newly discovered inversions considerably increase the complexity of plastome arrangements in the legume family, as indicated by a comparative analysis of genome structures across legumes (Supplementary Figure S3).

Large IR expansions and contractions are now known from numerous angiosperm lineages. In Fabaceae, the loss of the IRs in the IRLC papilionoids (including Cicereae, Fabeae, Galegeae s.l., Hedysareae, Millettieae p.p., Trifolieae, and a few allies like *Callerya, Glycyrrhiza*, and *Ononis*) represents the extreme end of the spectrum of plastome rearrangements in legumes (Wojciechowski et al., 2000). IR expansion in Fabaceae has been reported first from the IREC clade (including Ingeae and *Acacia* s.s.), which was named by a synapomorphic 13-kb IR expansion (Dugas et al., 2015; Wang et al., 2017a). In addition, IR-LSC junction shifts were found in two genera of IREC (Williams et al., 2015; Wang et al., 2017a). IR expansion into the SSC was also observed in the plastome of *Tylosema esculentum*, resulting in the duplication of the complete *ycf1* gene (Kim and Cullis, 2017). Here, we show that IR-SC junction shifts also affect *Bauhinia* s.l., except for *Bauhinia* itself (**Figure 2**). A double-strand break model (Goulding et al., 1996) and illegitimate recombination (Downie and Jansen, 2015; Blazier et al., 2016) may be causal for IR expansions and contractions in mimosoid plastomes (Wang et al., 2017a). The same mechanism may also underpin IR boundary shifts in Cercidoideae plastomes.

### Isomeric Plastome in *Tylosema* spp.

The 29-bp IR-associated inversion we observed in *T. fassoglensis* was also previously detected in some genistoids (Martin et al., 2014; Keller et al., 2017) as well as in a *Robinia* species (Schwarz et al., 2015), where it was thought to result from a flip-flop recombination event. As these 29-bp IRs, which lie at the 3′-ends of *trnS^GCU^* and *trnS^GGA^*, universally exist in almost all legume plastomes, it can be expected that the inversion between this pair of IRs might have occurred or may occur time and again in other legume plastomes through the same mechanism (Martin et al., 2014). Thus, flip-flop recombination may also explain the 29-bp IR-mediated 38-kb inversion in *T. fassoglensis*.

Interestingly, the plastome with this inversion appears to be an isomeric plastome (IPWI) that coexists with the canonical type (IPWC) in each individual of *Tylosema* (**Figure 3**). Isomeric plastomes, a result of flip-flop recombination, have been observed in several cupressophytes (Yi et al., 2013; Guo et al., 2014; Hsu et al., 2016; Qu et al., 2017b). Thus far, no isomeric plastomes have been reported in legumes, although two stable plastome configurations relating to a 45-kb inversion between a pair of imperfect repeats were found in different individuals of *Medicago truncatula* (Gurdon and Maliga, 2014). Here, we observed two arrangements of isomeric plastomes in four different *Tylosema* taxa. IPWI is the dominating conformation over IPWC in *T. fassoglensis* (the sequenced individual), based on both read-mapping and PCR results. PCR screens also confirmed the domination of IPWI in *T. fassoglensis* 1 as well as in *T. esculentum*. On the contrary, the IPWC dominates over IPWI in *T. fassoglensis* 2. In sum, our results illustrate that isomeric plastomes not only coexist in all of the examined taxa of *Tylosema*, but the proportions of their relative conformations may be individual-specific. These findings, therefore, provide essential, new insights into the complexity of plastomes in the legume family. Still, further study is needed to explore isomeric plastomes in other *Tylosema* species and legumes in general, and to clarify the molecular-evolutionary mechanisms and their relevance.

### Phylogenetic Relationships in Cercidoideae

**FIGURE 4:**
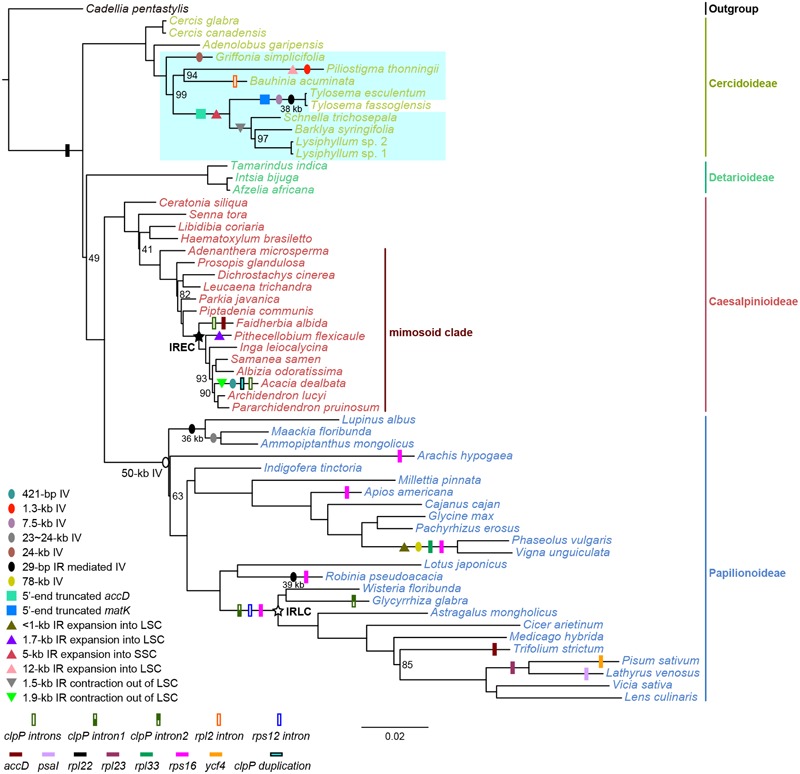
Maximum likelihood phylogeny reconstructed using a concatenated dataset of 77 plastid protein-coding genes and major plastome reconfigurations in legumes. The depicted ML tree was reconstructed from a concatenated dataset of 77 plastid protein-coding genes (CDS, Supplementary Figure S2). Species names are colored by their subfamily affiliation. Bootstrap support values of less than 100% are given at nodes. The scale bar indicates the mean number of nucleotide substitutions per site along a branch. As indicated in the legend at the bottom left, inversions (IV), functional, and physical gene losses, IR expansion/contraction, gene duplications, and intron losses are plotted onto branches using colored ovals, squares, triangles, and rectangles, respectively. Hollow circles and pentagons on nodes demarcate the most recent common ancestors of the 50-kb inversion clade, the IR-lacking clade (IRLC), and the IR expanding clade (IREC), respectively. Lengths of 29-bp IR mediated inversions are given below black ovals. A blue-shaded area highlights the plastome variations found in the species sequenced herein, while shown structural features other than these are summarized as reported by Bruneau et al. (1990); Schwarz et al. (2015), Choi and Choi (2017), and Wang et al. (2017a).

Several intergeneric relationships within Cercidoideae are hard to resolve (Bruneau et al., 2008; Sinou et al., 2009; LPWG, 2013, 2017). The sister relationship between *Adenolobus* and the remaining genera of Cercidoideae (except for the basal *Cercis*) were all weakly supported in these studies. Sinou et al. (2009) conducted a relatively dense-sampled phylogenetic study of this subfamily. In their work, the phylogenetic relationships of *Griffonia*, the Clade 1 of *Bauhinia* s.l. (represented by a clade of *Barklya* + *Lysiphyllum* + *Schnella* + *Tylosema* in our study), and the Clade 2 of *Bauhinia* s.l. (*Bauhinia* + *Piliostigma* herein) were unresolved. Our plastome data also failed to clarify the phylogenetic relationship of these three groups but allowed resolving most intergeneric relationships among the sampled genera (**Figure 4** and Supplementary Figure S2). Plastid phylogenomics has been successfully applied to resolve difficult relationships at the generic level (Ma et al., 2014; Givnish et al., 2015; Qu et al., 2017a; Zhang et al., 2017). However, the tree topology might not hold up when the second largest genus *Phanera* and four other genera of this subfamily are included in a phylogenetic survey of Cercidoideae. Also, plastid phylogenomics might only resolve a uniparental evolutionary line, not necessarily reflecting the full coalescent history (Wicke and Schneeweiss, 2015). Future studies with an improved, denser taxon sampling in combination with data of other genomic compartments may provide enhanced resolution of the relationships among the genera of this subfamily.

### Evolutionary Pattern of Plastome Variations in Legumes

The plastid *accD* gene encodes the β-carboxyl transferase subunit of acetyl-CoA carboxylase (ACCase). It is essential for plants but has been lost independently in at least six photosynthetic angiosperm lineages (Gurdon and Maliga, 2014). In some legume species, the plastid *accD* was reported to have been functionally transferred to the nucleus (Magee et al., 2010; Sabir et al., 2014), allowing the non-functionalization of the plastid copy. The highly divergent 5′-end of *accD* in *Pisum* (Doyle et al., 1995) may be as much a result of the non-functionalization of plastid *accD* as the 5′-end truncation of Cercidoideae *accD* we have reported here. The *matK* gene encodes an intron maturase, which has never been found to be a pseudogene or even absent from the plastome of a photosynthetic land plant (Zoschke et al., 2010; Wicke et al., 2011). We found that in *Tylosema*, the *matK* gene lacks more than 100 bp at its 5′-end although it still constitutes an intact open reading frame. As in other legumes, *Tylosema* retains the same set of group IIA-introns, which are usually associated with the *Matk* protein during splicing (Zoschke et al., 2010). In some Orobanchaceae, a truncation at *matK*’s 5′-end results in the use of an alternative start codon that restores the maturase function (Wolfe et al., 1992; Wicke et al., 2013). More research is needed to experimentally validate whether *accD* and *matK* are still functioning in *Tylosema*.

As mentioned earlier, many other structural plastome features have been reported as useful characters to support phylogenetic relationships in legumes (**Figure 4**). Here, we have shown that a shorter *accD* may be a synapomorphy of the clade containing *Barklya, Lysiphyllum, Schnella*, and *Tylosema*. A 5′-truncation of *matK* a 7.5-kb and a 29-bp IR-mediated 38-kb inversions characterize *Tylosema* plastomes. *Tylosema* and *Schnella* share a 5-kb IR expansion, which might have been ancestral to the entire clade, but which has lost from *Barklya* and *Lysiphyllum*. On the other hand, the 1.5-kb IR contraction we reported herein represents a synapomorphic character of the *Barklya*+*Lysiphyllum*+*Schnella* clade.

There are also many independent or parallel losses of genes or introns, inversions, and IR boundary shifts in legumes (**Figure 4**). In Cercidoideae, the 1.3 and 24-kb inversions are probably autapomorphies of *Piliostigma thonningii* and *Griffonia simplicifolia*, respectively, and the 12-kb IR expansion into the LSC may be another putative autapomorphy of *P. thonningii*. A denser sampling is needed to verify if these unusual plastome rearrangements are synapomorphic for certain lineages.

The loss of the *rpl2* intron has been reported in at least 18 angiosperm families and is thought to be a potentially useful phylogenetic character (Downie et al., 1991; Kelchner, 2002; Judd et al., 2008; Dong et al., 2016; Gu et al., 2016). In Fabaceae, the intron of *rpl2* lost several times independently in papilionoids, some *Bauhinia* species, and *P. thonningii* (Doyle et al., 1995; Lai et al., 1997; Sinou et al., 2009). Our study revealed that *Bauhinia acuminata* has lost the *rpl2* intron as well, thus corroborating the findings of Lai et al. (1997). However, we detected the *rpl2* intron in *P. thonningii*, a finding inconsistent with earlier reports (Sinou et al., 2009). Therefore, we believe that further research with an expanded sampling is urgently needed to determine the number of *rpl2* intron losses in legumes and to evaluate its phylogenetic relevance.

## Author Contributions

Y-HW, T-SY, D-ZL, and HW designed this research. Y-HW conducted the experiments and analyses. S-DZ collected some species and extracted DNA. J-JJ and S-YC assembled the plastomes. Y-HW, T-SY, and SW wrote the manuscript, and all authors revised it.

## Conflict of Interest Statement

The authors declare that the research was conducted in the absence of any commercial or financial relationships that could be construed as a potential conflict of interest.

## Footnotes

1 https://github.com/Kinggerm/GetOrganelle
