# Supplementary material for: Plastid Genome Evolution in the Early-Diverging Legume Subfamily Cercidoideae (Fabaceae)
Source: Front Plant Sci. 2018 Feb 8;9:138. doi: 10.3389/fpls.2018.00138 (PMC5812350; doi:10.3389/fpls.2018.00138)
Supplement: Supplementary file 1 [file Table_1.PDF]

Supplementary Material

Plastid genome evolution in the early-diverging legume subfamily Cercidoideae (Fabaceae)

Yin-Huan Wang, Susann Wicke, Hong Wang, Jian-Jun Jin, Si-Yun Chen, Shu-Dong Zhang, De-Zhu Li*, Ting-Shuang Yi*

*** Correspondence:** Ting-Shuang Yi tingshuangyi@mail.kib.ac.cn; De-Zhu Li dzl@mail.kib.ac.cn

Supplementary Table S1 Voucher information and type of materials of sampled Cercidoideae species.

Species	NCBI accession no.	Colleting no.	Herbarium	Type of materials
Barklya syringifolia	MF135594	Yi14483	KUN[1]	Fresh
Bauhinia acuminata	MF135595	Yi14407	KUN	Fresh
Griffonia simplicifolia	MF135596	766	UIH[2]	Silica gel-dried
Lysiphyllum binatum	MF135597	Yi14679	KUN	Fresh
Lysiphyllum hookeri	MF135601	Yi14678	KUN	Fresh
Piliostigma thonningii	MF135598	777	UIH	Silica gel-dried
Schnella trichosepala	MF135599	Sobrinho 2909	HUEFS[3]	Silica gel-dried
Tylosema fassoglensis	MF135600	OM1152	HJRAU	Total genomic DNA
Tylosema fassoglensis 1	NA	OM0602	HJRAU	Total genomic DNA
Tylosema fassoglensis 2	NA	OM1117	HJRAU	Total genomic DNA
Tylosema esculentum	NA	Yi15025	HJRAU	Total genomic DNA

[1] Herbarium of Kunming Institute of Botany, China; [2] Herbarium of University of Ibadan, Nigeria; [3] Universidade Estadual de Feira de Santana, Brazil; [4] Herbarium of University of Johnsonburg. NA, not available (these *Tylosema* samples were used only for PCR verification, their plastomes were not sequenced in this study).
