# Supplementary material for: Plastid Genome Evolution in the Early-Diverging Legume Subfamily Cercidoideae (Fabaceae)
Source: Front Plant Sci. 2018 Feb 8;9:138. doi: 10.3389/fpls.2018.00138 (PMC5812350; doi:10.3389/fpls.2018.00138)
Supplement: Supplementary file 2 [file Table_2.PDF]

Supplementary Material

Plastid genome evolution in the early-diverging legume subfamily Cercidoideae (Fabaceae)

Yin-Huan Wang, Susann Wicke, Hong Wang, Jian-Jun Jin, Si-Yun Chen, Shu-Dong Zhang, De-Zhu Li*, Ting-Shuang Yi*

*** Correspondence:** Ting-Shuang Yi tingshuangyi@mail.kib.ac.cn; De-Zhu Li dzl@mail.kib.ac.cn

Supplementary Table S2 Accession number and relevant reference of sampled legume plastomes obtained from GenBank.

Species	Accession no.	Reference
Acacia dealbata	NC_034985	Wang et al. (2017a)
Adenanthera microsperma	NC_034986	Wang et al. (2017a)
Adenolobus garipensis	KY806280	Wang et al. (2017b)
Afzelia africana	KX673213	Donkpegan et al. (2017)
Albizia odoratissima	NC_034987	Wang et al. (2017a)
Ammopiptanthus mongolicus	NC_034742	Feng et al. (2017)
Apios americana	NC_025909	-
Arachis hypogaea	NC_026676	-
Archidendron lucyi	NC_034988	Wang et al. (2017a)
Astragalus mongholicus	NC_029828	Lei et al. (2016)
Cadellia pentastylis	FC324	Wang et al. (2017a)
Cajanus cajan	NC_031429	-
Ceratonia siliqua	NC_026678	-
Cercis canadensis	KF856619	-
Cercis glabra	KY806281	Wang et al. (2017b)
Cicer arietinum	NC_011163	Jansen et al. (2008)
Dichrostachys cinerea	NC_035346	Wang et al. (2017a)
Faidherbia albida	NC_035347	Wang et al. (2017a)
Glycine max	NC_007942	Saski et al. (2005)
Glycyrrhiza glabra	NC_024038	Sabir et al. (2014)
Haematoxylum brasiletto	NC_026679	-
Indigofera tinctoria	NC_026680	-
Inga leiocalycina	NC_028732	Dugas et al. (2015)
Intsia bijuga	KX673214	Donkpegan et al. (2017)
Lathyrus venosus	NC_027080	-
Lens culinaris	NC_027152	-

Species	Accession no.	Reference
Leucaena trichandra	NC_028733	Dugas et al. (2015)
Libidibia coriaria	NC_026677	-
Lotus japonicus	NC_002694	Kato et al. (2000)
Lupinus albus	NC_026681	-
Maackia floribunda	NC_034774	Choi & Choi (2017)
Medicago hybrida	NC_027153	-
Millettia pinnata	NC_016708	Kazakoff et al. (2012)
Pachyrhizus erosus	NC_026682	-
Pararchidendron pruinosum	NC_035348	Wang et al. (2017a)
Parkia javanica	NC_034989	Wang et al. (2017a)
Phaseolus vulgaris	NC_009259	Guo et al. (2007)
Piptadenia communis	NC_034990	Wang et al. (2017a)
Pisum sativum	NC_014057	Magee et al. (2010)
Pithecellobium flexicaule	NC_034991	Wang et al. (2017a)
Prosopis glandulosa	NC_026683	-
Robinia pseudoacacia	NC_026684	-
Samanea samen	NC_034992	Wang et al. (2017a)
Senna tora	NC_030193	-
Tamarindus indica	NC_026685	-
Trifolium strictum	NC_025745	Sveinsson and Cronk (2014)
Tylosema esculentum	KX792933	Kim & Cullis (2017)
Vicia sativa	NC_027155	-
Vigna unguiculata	NC_018051	-
Wisteria floribunda	NC_027677	-

References:

Choi, I.S., and Choi, B.H. (2017). The distinct plastid genome structure of *Maackia fauriei* (Fabaceae: Papilionoideae) and its systematic implications for genistoids and tribe Sophoreae. *PLoS ONE* 12(4), e0173766.

Donkpegan, A.S.L., Doucet, J.-L., Migliore, J., Duminil, J., Dainou, K., Piñeiro, R*., et al.* (2017). Evolution in African tropical trees displaying ploidy-habitat association: The genus *Afzelia* (Leguminosae). *Mol. Phylogen. Evol.* 107(Supplement C)**,** 270-281.

Dugas, D.V., Hernandez, D., Koenen, E.J.M., Schwarz, E., Straub, S., Hughes, C.E*., et al.* (2015). Mimosoid legume plastome evolution: IR expansion, tandem repeat expansions, and accelerated rate of evolution in *clpP*. *Sci. Rep.* 5**,** 16958.

Feng, L., Gu, L.F., Luo, J., Fu, A.S., Ding, Q., Yiu, S.M*., et al.* 2017. Complete plastid genomes of the genus *Ammopiptanthus* and identification of a novel 23-kb rearrangement. *Conserv. Genet. Resour.* doi: 10.1007/s12686-017-0747-8.

Guo, X.W., Castillo-Ramirez, S., Gonzalez, V., Bustos, P., Fernandez-Vazquez, J.L., Santamaria, R.I*., et al.* 2007. Rapid evolutionary change of common bean (*Phaseolus vulgaris* L.) plastome, and the genomic diversification of legume chloroplasts. *BMC Genomics* 8:228. doi: 10.1186/1471-2164-8-228.

Jansen, R.K., Wojciechowski, M.F., Sanniyasi, E., Lee, S.B., and Daniell, H. (2008). Complete

plastid genome sequence of the chickpea (*Cicer arietinum*) and the phylogenetic distribution of *rps12* and *clpP* intron losses among legumes (Leguminosae). *Mol. Phylogen. Evol.* 48(3), 1204-1217.

Kato, T., Kaneko, T., Sato, S., Nakamura, Y., and Tabata, S. (2000). Complete structure of the chloroplast genome of a legume, *Lotus japonicus*. *DNA Res.* 7(6), 323-330.

Kazakoff, S.H., Imelfort, M., Edwards, D., Koehorst, J., Biswas, B., Batley, J., *et al.* (2012). Capturing the biofuel wellhead and powerhouse: The chloroplast and mitochondrial genomes of the leguminous feedstock tree *Pongamia pinnata*. *PLoS ONE* 7(12), e51687.

Kim, Y., and Cullis, C. (2017). A novel inversion in the chloroplast genome of marama (*Tylosema esculentum*). *J. Exp. Bot.* 68(8), 2065-2072.

Lei, W., Ni, D., Wang, Y., Shao, J., Wang, X., Yang, D., *et al.* (2016). Intraspecific and heteroplasmic variations, gene losses and inversions in the chloroplast genome of *Astragalus membranaceus*. *Sci. Rep.* 6, 21669.

Magee, A.M., Aspinall, S., Rice, D.W., Cusack, B.P., Semon, M., Perry, A.S., *et al.* (2010). Localized hypermutation and associated gene losses in legume chloroplast genomes. *Genome Res.* 20(12), 1700-1710.

Sabir, J., Schwarz, E., Ellison, N., Zhang, J., Baeshen, N.A., Mutwakil, M., *et al.* (2014). Evolutionary and biotechnology implications of plastid genome variation in the inverted-repeat-lacking clade of legumes. *Plant Biotechnol. J.* 12(6), 743-754.

Saski, C., Lee, S.-B., Daniell, H., Wood, T.C., Tomkins, J., Kim, H.-G., *et al.* (2005). Complete chloroplast genome sequence of *Glycine max* and comparative analyses with other legume genomes. *Plant Mol. Biol.* 59(2), 309-322.

Sveinsson, S., and Cronk, Q. 2014. Evolutionary origin of highly repetitive plastid genomes within the clover genus (*Trifolium*). *BMC Evol. Biol.* 14:228. doi: 10.1186/s12862-014-0228-6.

Wang, Y.H., Qu, X.J., Chen, S.Y., Li, D.Z., and Yi, T.S. 2017a. Plastomes of Mimosoideae: structural and size variation, sequence divergence, and phylogenetic implication. *Tree Genet. Genom.* 13:41. doi: 10.1007/s11295-017-1124-1.

Wang, Y.H., Wang, H., Yi, T.S., and Wang, Y.H. 2017b. The complete chloroplast genomes of *Adenolobus garipensis* and *Cercis glabra* (Cercidoideae, Fabaceae). *Conserv. Genet. Resour.* doi: 10.1007/s12686-017-0744-y.
