# Supplementary material for: Plastid Genome Evolution in the Early-Diverging Legume Subfamily Cercidoideae (Fabaceae)
Source: Front Plant Sci. 2018 Feb 8;9:138. doi: 10.3389/fpls.2018.00138 (PMC5812350; doi:10.3389/fpls.2018.00138)
Supplement: Supplementary file 3 [file Table_3.PDF]

Supplementary Material

Plastid genome evolution in the early-diverging legume subfamily Cercidoideae (Fabaceae)

Yin-Huan Wang, Susann Wicke, Hong Wang, Jian-Jun Jin, Si-Yun Chen, Shu-Dong Zhang, De-Zhu Li*, Ting-Shuang Yi*

*** Correspondence:** Ting-Shuang Yi tingshuangyi@mail.kib.ac.cn; De-Zhu Li dzl@mail.kib.ac.cn

Supplementary Table S3 Read-mapping results for the verification of the isomeric plastomes with inverted (IPWI) and canonical (IPWC) arrangements at four corresponding regions in *Tylosema fassoglensis*.

	**Isomeric plastome with inverted arrangement (IPWI)**		**Isomeric plastome with canonical arrangement (IPWC)**	
	psbI-trnS^{GGA}*-ycf3*	*trnG*^{UCC}*-trnS*^{GCU}*-rps4*	*psbI-trnS*^{GCU}*-trnG*^{UCC}	*ycf3-trnS*^{GGA}*-rps4*
Region size (bp)	2676	2306	1653	3329
Assembled reads	18,573	14,853	8,779	23,702
Aligned length (bp)	5314	4170	2840	6579
Mean coverage (×)	1032.3	988.6	787.3	1059.2
Reads covering the 29-bp IRs	802	777	7	8
