# Supplementary material for: Plastid Genome Evolution in the Early-Diverging Legume Subfamily Cercidoideae (Fabaceae)
Source: Front Plant Sci. 2018 Feb 8;9:138. doi: 10.3389/fpls.2018.00138 (PMC5812350; doi:10.3389/fpls.2018.00138)
Supplement: Supplementary file 4 [file Table_4.PDF]

Supplementary Material

Plastid genome evolution in the early-diverging legume subfamily Cercidoideae (Fabaceae)

Yin-Huan Wang, Susann Wicke, Hong Wang, Jian-Jun Jin, Si-Yun Chen, Shu-Dong Zhang, De-Zhu Li*, Ting-Shuang Yi*

*** Correspondence:** Ting-Shuang Yi tingshuangyi@mail.kib.ac.cn; De-Zhu Li dzl@mail.kib.ac.cn

Supplementary Table S4 Primer pairs and PCR conditions for the verification of the isomeric plastomes with inverted (IPWI) and canonical (IPWC) arrangements at four corresponding regions in four *Tylosema* species.

	Species	**Isomeric plastome with inverted arrangement (IPWI)**		**Isomeric plastome with canonical arrangement (IPWC)**	
		psbI-trnS^{GGA}*-ycf3*	*trnG*^{UCC}*-trnS*^{GCU}*-rps4*	*psbI-trnS*^{GCU}*-trnG*^{UCC}	*ycf3-trnS*^{GGA}*-rps4*
Forward primer	*T. fassoglensis*	TCTATCTAACGATCCAGGACGT	TTTTACCACTAAACTATACCCGC	TCTATCTAACGATCCAGGACGT	GAATTCATTGTATGTACCTGTACCT
	T. fassoglensis 1	TCTATCTAACGATCCAGGACGT	TTTTACCACTAAACTATACCCGC	TCTATCTAACGATCCAGGACGT	TTTATCCTACTGCCTTTCGAGGG
	T. fassoglensis 2	TCTATCTAACGATCCAGGACGT	TTTTACCACTAAACTATACCCGC	TCTATCTAACGATCCAGGACGT	TCACCTTCAGCAAGCGTAGAT
	T. esculentum	TCTATCTAACGATCCAGGACGT	TTTTACCACTAAACTATACCCGC	TCTATCTAACGATCCAGGACGT	GAATTCATTGTATGTACCTGTACCT
Reverse primer	*T. fassoglensis*	GCCTCTTTTTCTCCTGAAGTTGTC	GGGATGAACAAAAGTCTCGAGCT	TTTTACCACTAAACTATACCCGC	CGAATMATTCCCCTCGCGTYTA
	T. fassoglensis 1	GCCTCTTTTTCTCCTGAAGTTGTC	GGGATGAACAAAAGTCTCGAGCT	TGCATTGTMCAAGAATTCGTAGT	ATCTCTTGMCCACTYTTTCCGG
	T. fassoglensis 2	GCCTCTTTTTCTCCTGAAGTTGTC	GGGATGAACAAAAGTCTCGAGCT	TTTTACCACTAAACTATACCCGC	ATCTCTTGMCCACTYTTTCCGG
	T. esculentum	GCCTCTTTTTCTCCTGAAGTTGTC	GGGATGAACAAAAGTCTCGAGCT	TTTTACCACTAAACTATACCCGC	CGAATMATTCCCCTCGCGTYTA
Condition	*T. fassoglensis*	94℃, 3min; 94℃, 1min; 53.5℃, 1min and 40sec; 72℃, 1min; 35 cycles	94℃, 3min; 94℃, 1min; 53.5℃, 1 min and 40sec; 72℃, 1.5min; 35 cycles	94℃, 2min; 94℃, 1min; 48℃, 10sec; 65℃, 2min; 40 cycles *	94℃, 2min; 94℃, 1min; 48℃, 10sec; 65℃, 2min; 40 cycles *
	T. fassoglensis 1	94℃, 3min; 94℃, 1min; 53.5℃, 1min and 40sec; 72℃, 1min; 35 cycles	94℃, 3min; 94℃, 1min; 53.5℃, 1 min and 40sec; 72℃, 1.5min; 35 cycles	94℃, 3min; 94℃, 1min; 51℃, 1 min and 40sec; 72℃, 45sec; 35 cycles	94℃, 3min; 94℃, 1min; 51℃, 1 min and 40sec; 72℃, 45sec; 35 cycles
	T. fassoglensis 2	94℃, 3min; 94℃, 1min; 53.5℃, 1min and 40sec; 72℃, 1min; 35 cycles	94℃, 2min; 94℃, 1min; 48℃, 10sec; 65℃, 2min; 40 cycles *	94℃, 2min; 94℃, 1min; 48℃, 10sec; 65℃, 2min; 40 cycles *	94℃, 2min; 94℃, 1min; 48℃, 10sec; 65℃, 2min; 40 cycles *
	T. esculentum	94℃, 3min; 94℃, 1min; 53.5℃, 1min and 40sec; 72℃, 1min; 35 cycles	94℃, 3min; 94℃, 1min; 53.5℃, 1 min and 40sec; 72℃, 1.5min; 35 cycles	94℃, 2min; 94℃, 1min; 48℃, 10sec; 65℃, 2min; 40 cycles *	94℃, 2min; 94℃, 1min; 50℃, 10sec; 65℃, 2min; 40 cycles *

Notes: Primer sequences are shown in 5' -> 3' orientation. All PCR conditions included an initial denaturation followed by several cycles of a denaturation, annealing and elongation (each reaction is separated by semicolon). Conditions marked with an asterisk means the temperature is ramping up between each annealing and elongation step.
