# Supplementary figures and images for: Plastid Genome Evolution in the Early-Diverging Legume Subfamily Cercidoideae (Fabaceae)
Source: Front Plant Sci. 2018 Feb 8;9:138. doi: 10.3389/fpls.2018.00138 (PMC5812350; doi:10.3389/fpls.2018.00138)

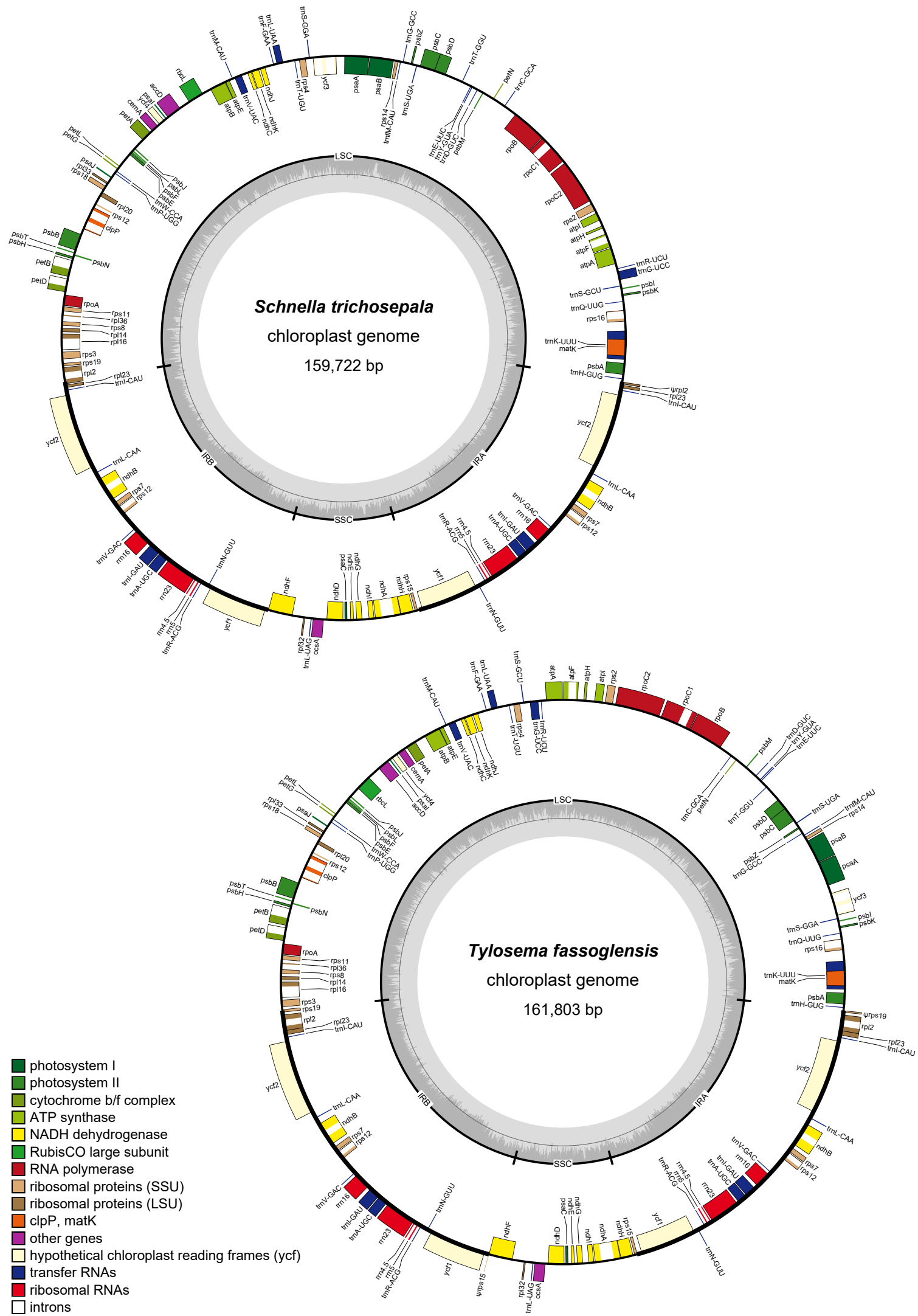

Supplementary Figure S1 Gene maps of the eight newly sequenced Cercidoideae plastomes.

Supplement: Supplementary file 5 [file Image_1.PDF]
