# Supplementary material for: Plastid Genome Evolution in the Early-Diverging Legume Subfamily Cercidoideae (Fabaceae)
Source: Front Plant Sci. 2018 Feb 8;9:138. doi: 10.3389/fpls.2018.00138 (PMC5812350; doi:10.3389/fpls.2018.00138)
Supplement: Supplementary file 6 [file Image_2.PDF]

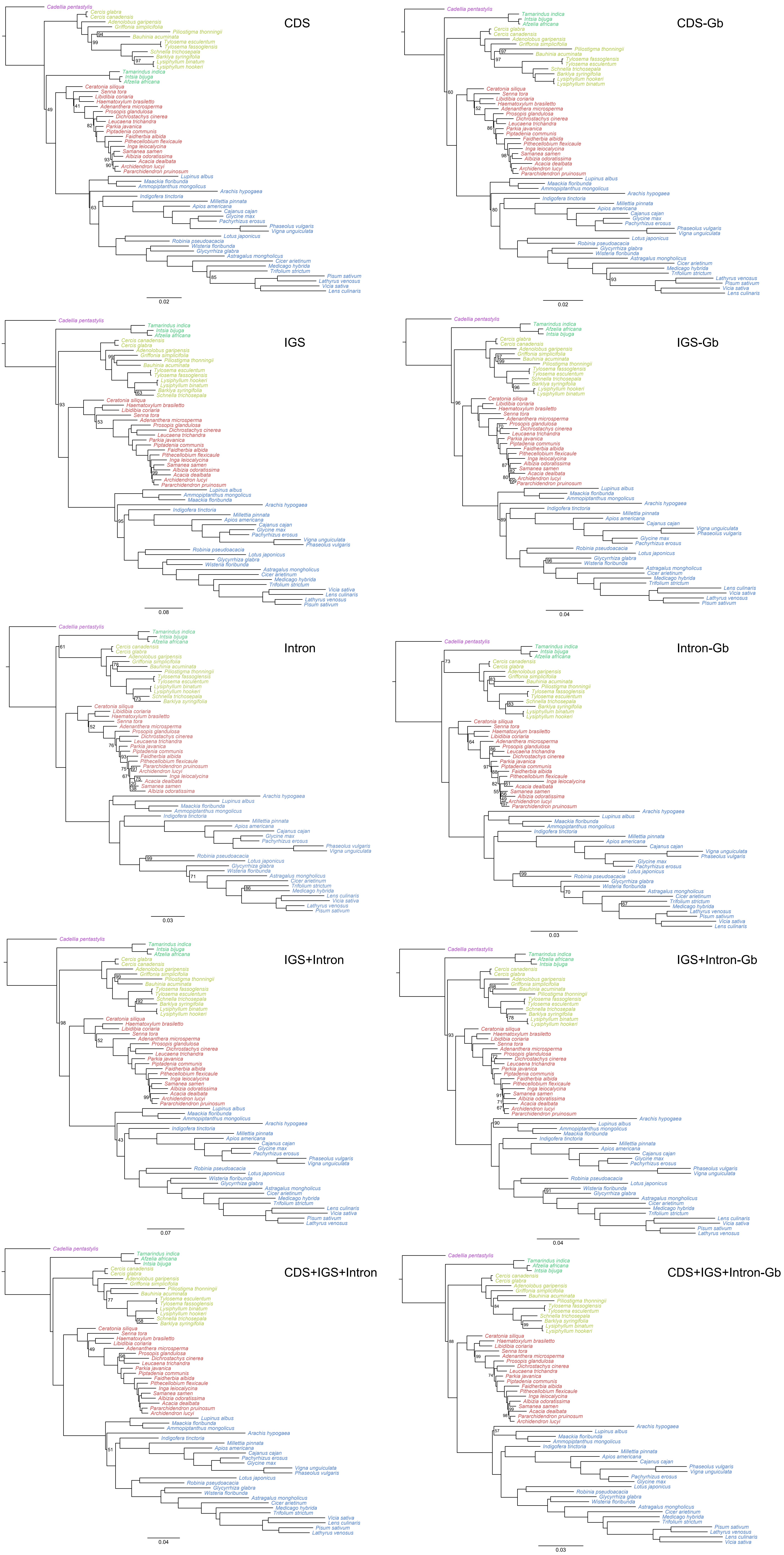

Supplementary Figure S2 Maximum likelihood phylogeny reconstructed using different plastid regions. At nodes with no maximum support, bootstrap support values are shown. Species names are colored by their subfamily affiliation: purple shows the outgroup, green the Detarioideae, yellow the Cercidoideae, red the Caesalpinioideae and blue the Papilionoideae.
