# Supplementary material for: Plastid Genome Evolution in the Early-Diverging Legume Subfamily Cercidoideae (Fabaceae)
Source: Front Plant Sci. 2018 Feb 8;9:138. doi: 10.3389/fpls.2018.00138 (PMC5812350; doi:10.3389/fpls.2018.00138)
Supplement: Supplementary file 7 [file Image_3.PDF]

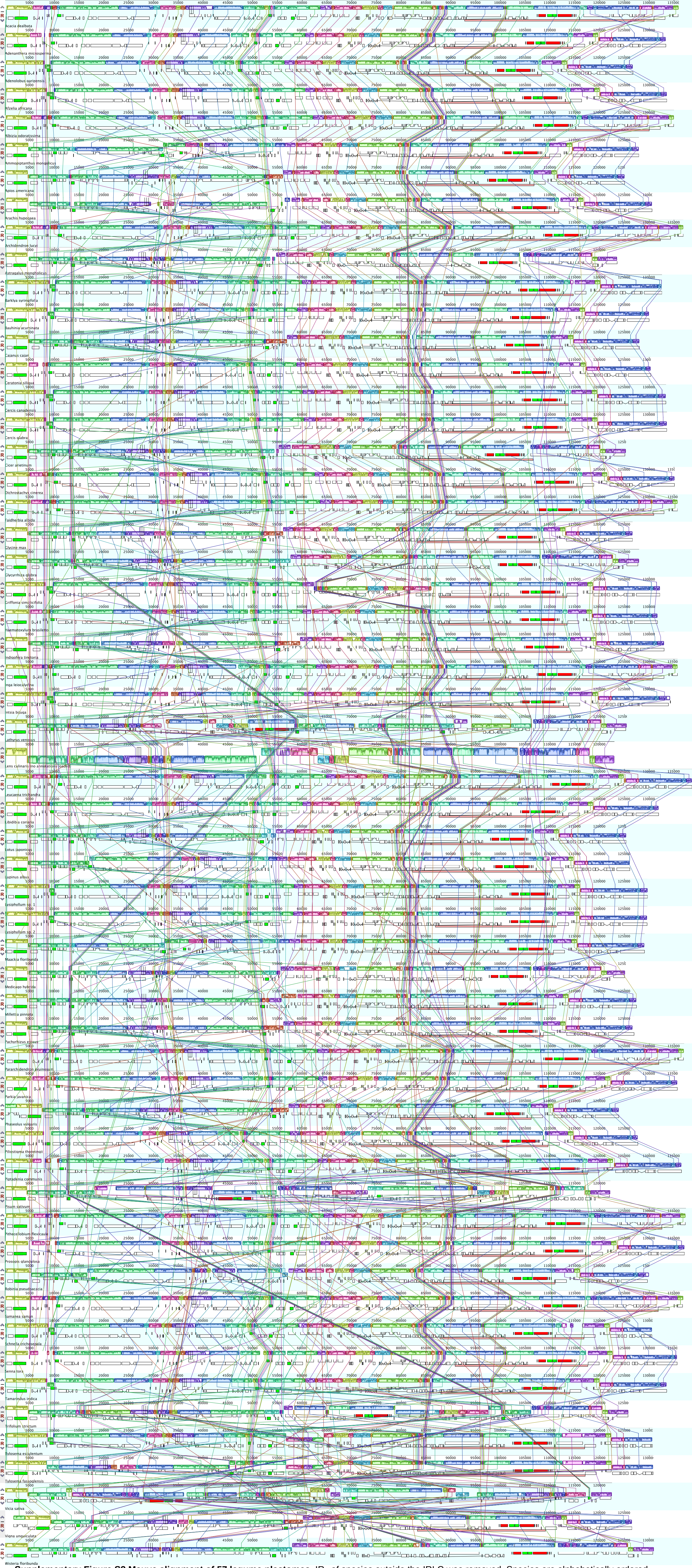

Supplementary Figure S3 Mauve alignment of 57 legume plastomes. IR_A of species outside the IRLC was removed. Species are alphabetically ordered.
